# *IL6*-174 G>C Polymorphism (rs1800795) Association with Late Effects of Low Dose Radiation Exposure in the Portuguese Tinea Capitis Cohort

**DOI:** 10.1371/journal.pone.0163474

**Published:** 2016-09-23

**Authors:** Paula Boaventura, Cecília Durães, Adélia Mendes, Natália Rios Costa, Inês Chora, Sara Ferreira, Emanuel Araújo, Pedro Lopes, Gilberto Rosa, Pedro Marques, Paulo Bettencourt, Inês Oliveira, Francisco Costa, Isabel Ramos, Maria José Teles, João Tiago Guimarães, Manuel Sobrinho-Simões, Paula Soares

**Affiliations:** 1 IPATIMUP—Institute of Molecular Pathology and Immunology of the University of Porto, Rua Júlio Amaral de Carvalho 45, 4200–135 Porto, Portugal; 2 i3S—Instituto de Investigação e Inovação em Saúde, Universidade do Porto, Portugal, Rua Alfredo Allen 208, 4200–135 Porto, Portugal; 3 Hospital of S. João, Alameda Prof. Hernani Monteiro, 4200–319 Porto, Portugal; 4 Faculty of Medicine of the University of Porto, Alameda Prof. Hernani Monteiro, 4200–319 Porto, Portugal; University of South Alabama Mitchell Cancer Institute, UNITED STATES

## Abstract

Head and neck cancers, and cardiovascular disease have been described as late effects of low dose radiation (LDR) exposure, namely in tinea capitis cohorts. In addition to radiation dose, gender and younger age at exposure, the genetic background might be involved in the susceptibility to LDR late effects. The -174 G>C (rs1800795) SNP in *IL6* has been associated with cancer and cardiovascular disease, nevertheless this association is still controversial. We assessed the association of the *IL6*-174 G>C SNP with LDR effects such as thyroid carcinoma, basal cell carcinoma and carotid atherosclerosis in the Portuguese tinea capitis cohort. The *IL6*-174 G>C SNP was genotyped in 1269 individuals formerly irradiated for tinea capitis. This sampling group included thyroid cancer (n = 36), basal cell carcinoma (n = 113) and cases without thyroid or basal cell carcinoma (1120). A subgroup was assessed for atherosclerosis by ultrasonography (n = 379) and included matched controls (n = 222). Genotypes were discriminated by real-time PCR using a TaqMan SNP genotyping assay. In the irradiated group, we observed that the CC genotype was significantly associated with carotid plaque risk, both in the genotypic (OR = 3.57, CI = 1.60–7.95, *p-value* = 0.002) and in the recessive (OR = 3.02, CI = 1.42–6.42, *p-value* = 0.004) models. Irradiation alone was not a risk factor for carotid atherosclerosis. We did not find a significant association of the *IL6*-174 C allele with thyroid carcinoma or basal cell carcinoma risk. The *IL6*-174 CC genotype confers a three-fold risk for carotid atherosclerotic disease suggesting it may represent a genetic susceptibility factor in the LDR context.

## Introduction

Head and neck cancers are considered late effects of low dose radiation (LDR) exposure, namely in the former tinea capitis patients who were submitted to scalp irradiation to induce epilation [1]. We have found a high prevalence of basal cell carcinoma (BCC) [2] and of thyroid carcinoma (TC) [3] in the Portuguese tinea capitis cohort, in accordance with what has been previously described in similar cohorts [4–6]. Recently, in the Israeli tinea capitis cohort, scalp irradiation has been shown as an independent risk factor for the development of carotid stenosis due to the formation of atherosclerotic plaques in adults [7].

The irradiation dose, gender and younger age at irradiation have been associated with higher risk of TC and BCC in such cohorts [2,4,6,8]. We hypothesize the genetic background may also be involved in the susceptibility to develop these radiation-associated late effects [9].

Interleukin 6 (IL6) is a cytokine with a regulatory role in cell proliferation, differentiation and the balance between pro-inflammatory and anti-inflammatory pathways [10]. It has been implicated in cardiovascular disease [11,12] and neoplastic disease [10,13]. The promoter of *IL6* contains several SNPs, of which the -174 G>C is the most widely studied for its influence in various cancers [14]. The association of this SNP with different cancers has been extensively analyzed, nevertheless, some of the associations are still inconclusive or in opposite trends for different types of cancer [14]. Jiao and colleagues have shown the -174 G>C SNP was not associated with lung cancer as previously described [15]. Contrarily to what has been suggested, Yu and colleagues reported a lack of association with breast cancer in a meta-analysis involving more than 25000 subjects [16]. Similar inconsistency in risk estimates is found for BCC, with some studies reporting no association [17] and other reporting association of a different *IL6* SNP (*IL6*-597 G>A) [18]. Regarding TC, Schulte and colleagues found a higher C allele frequency, reaching significance only for the papillary variant [19].

Cardiovascular disease has also been associated with the SNP, namely coronary heart disease and carotid atherosclerosis (20). However, in other studies, namely in ischemic stroke [20] and cardiac arrhythmias in children [12], no association was found.

In our study we assessed the association of the *IL6*-174 G>C SNP with TC, BCC and carotid atherosclerosis in the Portuguese tinea capitis cohort. To the best of our knowledge, there are no susceptibility studies of the *IL6*-174 G>C SNP in cancer and cardiovascular disease susceptibility in the LDR exposure context.

## Materials and Methods

### Study population

From 2006 to 2012 we have clinically observed 1375 individuals from a cohort of 5356 individuals submitted, in childhood, to X-ray scalp epilation for tinea capitis treatment, as previously described [3]. Briefly, from the 1375 individuals that agreed to a clinical appointment, we registered the TC and BCC cases retrospectively diagnosed, and the new cases prospectively diagnosed through thyroid scans, fine needle aspiration for suspicious nodules and surgery for TC, and surgery of head and neck suspicious lesions for BCC. Of these individuals, 38 had TC (2.8%) and 113 had BCC (8.2%).

From the 1375 individuals, we randomly selected 690 (52.5%) to whom we were able to propose a Doppler exam, in a second evaluation directed to carotid atherosclerosis diagnosis. These selected individuals did not significantly differ from the ones not selected concerning gender, age and radiation dose. The individuals were contacted by phone and we were able to include in the present study 379 (55.0%). In addition to the Doppler exam, information about smoking habits (smoking pack years), diabetes, and hypertension was obtained. An inaccessible phone number was the main reason for not reaching the 311 individuals that were not included.

The control group (non-irradiated) comprises individuals invited to participate at the moment of the clinical appointment of the irradiated participants, and to whom the same protocol was applied. This group (n = 222) is composed mainly of the participants’ spouses (90%) and friends (10%). The control group is smaller than the irradiated group because not all irradiated participants were accompanied by a chaperone (several were divorced or widowed), and not bringing a chaperone was not an exclusion criterion for participation. The exclusion criteria were age other than 50–75 years old and exposure to radiation, except for diagnostic purposes. Irradiated individuals and controls were submitted to the same protocol and all the physicians and technicians involved in the study were blinded to the participants’ past irradiation history. The study was approved by the ARS-Norte ethical committee and all the participants signed an informed consent form. The demographic and clinicopathological features of these groups are described in Table 1 and S1 File.

**Table 1:** Demographic and clinicopathological characteristics of the total cohort, thyroid carcinoma, basal cell carcinoma and individuals examined by Doppler ultrasound.

Groups		Gender	Age	Hypertension	Diabetes	Smoking pack years
		female:male (%)	years	n(%)	n(%)	n(%)
Irradiated, total cohort	n	759:511	1269	---	---	---
		(59.9:40.1)	58.5 ± 4.4			
Irradiated, thyroid carcinoma	n	28:8	36	---	---	---
		(77.8:22.2)	57.9 ± 3.4			
Irradiated, basal cell carcinoma	n	76:37	113	---	---	---
		(67.3:32.7)	59.7 ± 4.7			
Irradiated, with Doppler	n	221:158	379	375	375	366
		(58.3:41.7)	63.0 ± 3.9	216 (57.6)	89 (23.7)	10.4 ± 21.8
Non-irradiated, with Doppler	n	121:101	222	220	219	217
		(54.5:45.5)	62.2 ± 5.5	104 (47.3)	33 (15.1)	11.3 ± 21.3

### Doppler ultrasound

A B-mode ultrasound imaging of carotid arteries for intima media thickness (IMT) and stenosis evaluation was performed. The ultrasonography was accomplished with a Philips iU22 device, using a linear transducer of 8–9 MHz according to a standardized protocol [21]. Briefly, the patients were placed in a supine position in a dark quiet room, and the right and left carotid arteries were examined in supine midline position. IMT was measured in the longitudinal plan at the point of maximum thickness on the far wall of the common carotid artery (values ≥1mm were considered as increased IMT [21]. Plaque presence and carotid stenosis (<30% or ≥30%) [7] were also assessed. The exams were performed by experienced radiologists (manuscript authors IO, AS, FC).

### DNA extraction and SNP genotyping

Whole blood from irradiated and non-irradiated individuals was collected at Hospital of S. João, Porto, Portugal, directly into 3mL EDTA tubes (Vacutest Kima K_3_ EDTA v5.4mg, Arzergrande, Italy). Genomic DNA was extracted using Miller’s DNA extracting method [22] with an additional chloroform step. All DNA samples are stored at -80°C at Ipatimup/i3S. For the present study, there was genomic DNA available from 1269 of the 1375 (92.3%) individuals observed in the first phase of the study (comprising 36 TC, 113 BCC and 1120 cases without TC or BCC). From these 1269 individuals, 379 were submitted to Doppler examinations in the second part of the study. Additionally, we included 222 non-irradiated individuals for comparison, not observed in the first part of the study.

SNP *IL6*-174 G>C (rs1800795) was genotyped using a TaqMan Custom-Designed SNP Genotyping Assay (Applied Biosystems, Carlsbad, USA). PCR amplification and allelic discrimination were performed according to product specifications with the ABI 7500 Fast real-time PCR system (Applied Biosystems, Carlsbad, USA). Irradiated and non-irradiated samples were randomized during genotyping and 10% were genotyped in duplicate to assess the genotyping error rate (genotype concordance was 100%).

### Statistical analysis

Genotype frequencies for the *IL6*-174 SNP were obtained using SPSS 23 (IBM SPSS Statistics). Compliance of alleles with the Hardy-Weinberg equilibrium was measured at the level of the control population using a χ^2^ test (level of significance set to *p-value* <0.05). Differences between the irradiated and non-irradiated groups regarding plaque presence, IMT and stenosis were assessed using a χ^2^ test (*p-value* <0.05).

Comparison of genotype frequencies between groups was assessed by unconditional logistic regression (level of significance set to *p-value* <0.05) with SPSS 23. The models included the adjustment variables gender and age for the TC and BCC association analysis, and gender, age, diabetes *status*, hypertension *status* and smoking habits for the atherosclerosis association analysis (*p-values* are reported in S1–S4 Tables).

Odds ratios (OR) with respective confidence intervals (95% CI) were calculated considering the genotypic, dominant and recessive models of inheritance. The adjustment for multiple testing was performed by the false discovery rate (FDR) method [23]. All power analyses were performed using CATS for a one-stage study.

## Results

The genotype frequencies of the *IL6*-174 SNP did not deviate significantly from those expected under the Hardy-Weinberg equilibrium (*p-value* = 0.771). The genotyping success rate was 100% in all groups and the genotyping error rate was 0%.

The distribution of the genotypes in the total cohort (n = 1269) was as follows: GG, n = 499 (39.3%); GC, n = 597 (47.1%); CC, n = 173 (13.6%). We did not find significant association of the *IL6*-174 C allele with TC or BCC, in the dominant and in the recessive models (adjusted for gender and age) (Table 2, S1 File and S1 Table).

**Table 2:** Genotypic frequencies and association of the *IL6*-174 SNP with thyroid carcinoma and basal cell carcinoma (adjusted for gender an age).

Locus/genotype	Without TC	With TC			Without BCC	With BCC		
*IL6*-174 G>C	n (%)	n (%)	OR (95% CI)	*p-value*	n (%)	n (%)	OR (95% CI)	*p-value*
	n = 1233	n = 36			n = 1156	n = 113		
GG	484 (39.3)	15 (41.7)	1.00^a^		462 (40.0)	37 (32.7)	1.00^a^	
GC	580 (47.0)	17 (47.2)	0.94 (0.46–1.91)	0.867	540 (46.7)	57 (50.4)	1.30 (0.84–2.00)	0.235
CC	169 (13.7)	4 (11.1)	0.76 (0.24–2.31)	0.622	154 (13.3)	19 (16.8)	1.51 (0.84–2.70)	0.172
Dominant model (*C carrier vs GG*^b^)	749 (60.8) / 484 (39.2)	21 (58.3) / 15 (41.7)	0.90 (0.46–1.76)	0.757	694 (60.0) / 462 (40.0)	76 (67.3) / 37 (32.7)	1.35 (0.89–2.03)	0.157
Recessive model (*CC vs G carrier*^b^)	169 (13.7) / 1064 (86.3)	4 (11.1) / 32 (88.9)	0.78 (0.27–2.24)	0.644	154 (13.3) / 1002 (86.7)	19 (16.8) / 94 (83.2)	1.29 (0.77–2.19)	0.336

^a^ reference value.

^b^ reference genotype.

In the subgroup submitted to carotid atherosclerosis evaluation we considered separately three different variables, retrieved from the Doppler exams: i) plaque presence; ii) IMT and iii) percentage of carotid stenosis. The analysis of irradiated *vs* non-irradiated cases according to these variables showed no significant differences in carotid atherosclerosis: 45% (171/380) *vs* 42.3% (94/222) for plaque presence (*p-value* = 0.526), 10.6% (40/377) *vs* 9.5% (21/222) for stenosis (*p-value* = 0.653), and 13.9% (53/380) *vs* 9.5% (21/222) for high IMT (*p-value* = 0.106).

In the whole cohort, independently of radiation exposure, we did not observe statistically significant differences in the frequencies of *IL6*-174 genotypes according to carotid plaque presence, increased IMT or stenosis ≥30% (in the dominant or the recessive models) after adjustment for gender, age, hypertension, diabetes, and smoking habits (Table 3 and S2 Table). To evaluate the effect of the radiation exposure we analysed separately the irradiated and non-irradiated groups (Table 4, S3 Table and S4 Table).

**Table 3:** Genotypic frequencies and association of the *IL6*-174 SNP with presence of plaques, increased IMT, and degree of stenosis (adjusted for gender, age, hypertension, diabetes, and smoking habits).

**Locus/genotype**	**Without Plaques**	**With Plaques**		
	**n (%)**	**n (%)**	**OR (95% CI)**	***p-value***
***IL6*-174 G>C**	n = 326	n = 251		
GG	137 (42.0)	93 (37.1)	1.00^a^	
GC	162 (49.7)	123 (49.0)	1.12 (0.77–1.63)	0.549
CC	27 (8.3)	35 (13.9)	2.18 (1.19–3.97)	0.011^c^
Dominant model (*C carrier vs GG*^b^)	189 (58.0) / 137 (42.0)	158 (62.9) / 93 (37.1)	1.26 (0.88–1.80)	0.200
Recessive model (*CC vs G carrier*^b^)	27 (8.3) / 299 (91.7)	35 (13.9) / 216 (86.1)	2.04 (1.16–3.59)	0.013^c^
	**Normal IMT**	**Increased IMT**		
	**n (%)**	**n (%)**	**OR (95% CI)**	***p-value***
***IL6*-174 G>C**	n = 505	n = 72		
GG	201 (39.8)	29 (40.3)	1.00^a^	
GC	254 (50.3)	31 (43.1)	0.78 (0.44–1.38)	0.396
CC	50 (9.9)	12 (16.7)	1.76 (0.80–3.86)	0.162
Dominant model (*C carrier vs GG*^b^)	304 (60.2) / 201 (39.8)	43 (59.7) / 29 (40.3)	0.93 (0.55–1.57)	0.783
Recessive model (*CC vs G carrier*^b^)	50 (9.9) / 455 (90.1)	12 (16.7) / 60 (83.3)	2.00 (0.96–4.17)	0.064
	**Stenosis <30%**	**Stenosis ≥30%**		
	**n (%)**	**n (%)**	**OR (95% CI)**	***p-value***
***IL6*-174 G>C**	n = 515	n = 59		
GG	210 (40.8)	18 (30.5)	1.00^a^	
GC	250 (48.5)	34 (57.6)	1.69 (0.91–3.13	0.097
CC	55 (10.7)	7 (11.9)	1.65 (0.63–4.28)	0.308
Dominant model (*C carrier vs GG*^b^)	305 (59.2) / 210 (40.8)	41 (69.5) / 18 (30.5)	1.68 (0.92–3.05)	0.090
Recessive model (*CC vs G carrier*^b^)	55 (10.7) / 460 (89.3)	7 (11.9) / 52 (88.1)	1.21 (0.51–2.88)	0.673

^a^ reference value.

^b^ reference genotype.

^c^ non-significant values after FDR correction.

IMT–intima media thickness.

**Table 4:** Genotypic frequencies and association of the *IL6*-174 SNP with presence of plaques, increased IMT and degree of stenosis, in the irradiated and non-irradiated individuals (adjusted for gender, age, hypertension, diabetes, and smoking habits).

**Locus/genotype**	**Non-irradiated**				**Irradiated**			
	**Without plaques**	**With plaques**	** **	** **	**Without plaques**	**With plaques**	** **	** **
	**n (%)**	**n (%)**	**OR (95% CI)**	***p-value***	**n (%)**	**n (%)**	**OR (95% CI)**	***p-value***
***IL6*-174 G>C**	n = 124	n = 90		*** ***	n = 202	n = 161		*** ***
GG	49 (39.5)	41 (45.6)	1.00^a^		88 (43.6)	52 (32.3)	1.00^a^	
GC	60 (48.4)	39 (43.3)	0.89 (0.47–1.67)	0.714	102 (50.5)	84 (52.2)	1.34 (0.84–2.14)	0.227
CC	15 (12.1)	10 (11.1)	1.11 (0.41–2.96)	0.843	12 (5.9)	25 (15.5)	**3.57 (1.60–7.95)**	**0.002**
Dominant model (*C carrier vs GG*^b^)	75 (60.5) / 49 (39.5)	49 (54.4) / 41 (45.6)	0.93 (0.51–1.69)	0.806	114 (56.4) / 88 (43.6)	109 (67.7) / 52 (32.3)	1.57 (1.00–2.46)	0.052
Recessive model (*CC vs G carrier*^b^)	15 (2.1) / 109 (87.9)	10 (11.1) / 80 (88.9)	1.27 (0.46–2.98)	0.734	12 (5.9) / 190 (94.1)	25 (15.5) / 136 (84.5)	**3.02 (1.42–6.42)**	**0.004**
								** **
	**Normal IMT**	**Increased IMT**		** **	**Normal IMT**	**Increased IMT**		** **
	**n (%)**	**n (%)**	**OR (95% CI)**	***p-value***	**n (%)**	**n (%)**	**OR (95% CI)**	***p-value***
***IL6*-174 G>C**	n = 193	n = 21		*** ***	n = 312	n = 51		*** ***
GG	77 (39.9)	13 (61.9)	1.00^a^		124 (39.7)	16 (31.4)	1.00^a^	
GC	95 (49.2)	4 (19.0)	0.25 (0.076–0.821)	0.022^c^	159 (51.0)	27 (52.9)	1.21 (0.60–2.44)	0.598
CC	21 (10.9)	4 (19.0)	1.47 (0.40–5.45)	0.567	29 (9.3)	8 (15.7)	1.92 (0.69–5.31)	0.210
Dominant model (*C carrier vs GG*^b^)	116 (60.1) / 77 (39.9)	8 (38.1) / 13 (61.9)	0.43 (0.16–1.11)	0.082	188 (60.3) / 124 (39.7)	35 (68.6) /16 (31.4)	1.32 (0.67–2.59)	0.421
Recessive model (*CC vs G carrier*^b^)	21 (10.9) / 172 (89.1)	4 (19.0) / 81.0)	2.54 (0.71–9.0)	0.151	29 (9.3) / 283 (90.7)	8 (15.7) / 43 (84.3)	1.71 (0.68–4.31)	0.253
								
	**Stenosis <30%**	**Stenosis ≥30%**		** **	**Stenosis <30%**	**Stenosis ≥30%**		** **
	**n (%)**	**n (%)**	**OR (95% CI)**	***p-value***	**n (%)**	**n (%)**	**OR (95% CI)**	***p-value***
***IL6*-174 G>C**	n = 193	n = 21		*** ***	n = 322	n = 38		*** ***
GG	80 (41.5)	10 (47.6)	1.00^a^		130 (40.4)	8 (21.1)	1.00^a^	
GC	90 (46.6)	9 (42.9)	1.03 (0.38–2.81)	0.960	160 (49.7)	25 (65.8)	2.58 (1.11–6.07)	0.030^c^
CC	23 (11.9)	2 (9.5)	1.11 (0.21–5.80)	0.913	32 (9.9)	5 (13.2)	2.47 (0.72–8.45)	0.151
Dominant model (*C carrier vs GG*^b^)	113 (58.5) / 80 (41.5)	11 (52.4) / 10 (47.6)	1.04 (0.40–2.71)	0.938	192 (59.6) / 130 (40.4)	30 (78.9) / 8 (21.1)	2.56 (1.11–5.91)	0.028^c^
Recessive model (*CC vs G carrier*^b^)	23 (11.9) / 170 (88.1)	2 (9.5) / 19 (90.5)	1.08 (0.222–5.29)	0.922	32 (9.9) / 290 (90.1)	5 (13.2) / 33 (86.8)	1.31 (0.46–3.79)	0.613

^a^ reference value.

^b^ reference genotype.

^c^ non-significant values after FDR correction.

IMT–intima media thickness.

Power calculations conducted before the study indicated that, in the irradiated group, there was more than 80% power to detect significant associations of OR between 1.3 and 1.8 [MAF = 35% [24]; OR = 1.3–1,8; plaque prevalence = 42.0%, and case/control = 160/200]. In the irradiated group we observed that the CC genotype was significantly associated with carotid plaque presence in the genotypic (OR = 3.57, CI = 1.60–7.95, *p-value* = 0.002) and in the recessive (OR = 3.02, CI = 1.42–6.42, *p-value* = 0.004) models. The significance was retained after FDR multiple test correction. A post-hoc power analysis showed, for an OR of 3.02 (recessive model), the power to detect a significant association is 100%.

In the non-irradiated group, we did not observe statistically significant differences in the frequencies of genotypes or alleles between cases with or without carotid plaques.

No other significant associations were found in the remaining variables from the Doppler exams—IMT and stenosis—evaluated in the present study.

## Discussion

We have evaluated the association of the *IL6*-174 G>C SNP with late effects of LDR (TC, BCC and carotid atherosclerotic disease) in a cohort of individuals irradiated in childhood for tinea capitis treatment. We found that the *IL6*-174 CC genotype confers a three-fold risk for carotid atherosclerotic disease compared with non-irradiated individuals.

There are few reports on TC or BCC and *IL6* polymorphisms [17,18,25,26], and none of which in the radiation exposure context. Regarding TC, Cil and colleagues found *IL6*-174 GG genotype conferred increased risk, albeit only with the concomitant presence of the *IL10*-1082 G allele (GG+AG genotypes) (OR 1.75, 95% CI 1.00–3.05, p = 0.049) [25]. Ozgen and colleagues [26] suggested the *IL6*-174 G>C SNP could play a role on TC risk although they did not find an effective role as a prognostic factor. Schulte and colleagues [19] found higher C allele frequencies in all types of TC, reaching statistical significance in papillary TC. Overall, these authors showed the *IL6*-174 G>C polymorphism could play a role in TC, even though the data are not very conclusive.

In our irradiated the frequency of the C allele in the control group (37.2%) was similar to that observed in the control group of a previous study on thyroid disease (34.6%) [24]. We did not find a significant association of this polymorphism with TC or a significant increase in the C allele frequency. Regarding BCC, Vogel and colleagues [17] reported no association of *IL6*-174 G>C with BCC, whereas Wilkening and colleagues found a protective effect of genotype GA in *IL6*-597 G>A (OR 0.64, 95% CI 0.49–0.84) [18]. Similarly to what we observed for TC, there was no significant association of *IL6*-174 G>C with BCC. A meta-analysis comprising 44735 cancer patients and 60747 controls reported that, in overall, the *IL6*-174 G/C polymorphism was not significantly associated with cancer [27]. However, cancer risk was increased for individuals with the CC genotype in African populations (OR = 1.83, 95% CI 1.26–2.67, p-value = 0.002) but not in Caucasian populations (OR = 1.00, 95% CI 0.92–1.08, p-value = 0.938) [27].

Several studies have reported *IL6* polymorphisms association with cardiovascular disease [28–32] whereas others have found no association [12,20,33], therefore, the current concept of an *IL6* polymorphism as a cardiovascular risk factor is still under dispute [29]. Carotid atherosclerosis can be evaluated either through IMT measurement [34–36], carotid stenosis [34,37] or plaque presence [34,36,37], thus, we assessed independently these three variables. In a similar cohort (Israeli tinea capitis cohort), Shai and colleagues evaluated IMT and stenosis degree, considering ≥30% as the cut-off for the presence of stenosis [7], therefore we also adopted this cut-off.

Considering the data on *IL6* polymorphisms association with cardiovascular disease are still controversial, and that we did not find reports in the context of radiation exposure, we analyzed together, as a first approach, the irradiated and non-irradiated groups. Including the full data set, we did not find a statistically significant association between the *IL6*-174 SNP and carotid plaque presence, increased IMT or stenosis ≥30%. Our data do not confirm the findings of Rundek and colleagues who observed, in a sample of 87 subjects, a higher IMT in those presenting the GG genotype [38], or the findings of Rauramaa and colleagues who also found an equivalent association in a sample of 109 subjects [30]. Nevertheless, our data are in agreement with the absence of association between IMT and the *IL6*-174 G>C SNP reported by Chumaeva and colleagues in a larger sample of 1673 subjects [11]. Moreover, a meta-analysis of 50 studies involving more than 34000 subjects, did not find an association between any allele of the *IL6*-174 G>C SNP and atherosclerosis [39].

In our cohort, radiation exposure was not a risk factor for carotid atherosclerosis, since there was no difference in the evaluated outcome when comparing irradiated with non-irradiated groups. Yet, we found that the CC genotype in *IL6*-174 G>C conferred a three-fold risk of carotid plaque development in the group exposed to radiation. To the best of our knowledge, this is the first study implying the *IL6*-174 G>C polymorphism in atherosclerosis susceptibility in the irradiation context. Considering this irradiation treatment *per se* has been described by others as an independent risk factor for carotid atherosclerosis [7], our study suggests that individuals carrying the susceptibility genotype and submitted to LDR in childhood should be followed for carotid atherosclerosis early detection. This is particularly important because LDR is the range of dose frequently experienced in routine medical exams. In addition to age at irradiation and irradiation dose, genetic susceptibility has been pointed out as a possible risk factor for long-term side effects of radiation treatment. Our work supports this assumption showing the association of the C allele in *IL6*-174 G>C SNP with carotid atherosclerosis in the irradiation context.

One limitation of our study is that we did not re-evaluate and perform Doppler exams to all the irradiated individuals formerly observed, resulting in a smaller subgroup of 379 individuals for carotid atherosclerosis assessment. Nevertheless, no significant differences were observed between these individuals and the ones that were not observed for atherosclerotic disease concerning gender, irradiation dose, and previous cardiovascular or cerebrovascular disease. The only difference was younger age at irradiation, with 39.1% of the 379 individuals irradiated at a younger age (≤5 years of age) comparing with the 30.1% in the group that was not observed. This difference was also observed in our previous study [1] as the individuals irradiated at a younger age were also younger at the beginning of the study, therefore more agreeable to attend to the clinical appointment.

In summary, in the present study we did not find a significant association of the *IL6*-174 G>C polymorphism with head and neck cancer (TC and BCC) as reported by others. In contrast, we found a significant association between the polymorphism and atherosclerosis, through plaque presence, in the irradiation context. This suggests the *IL6*-174 G>C polymorphism may be a genetic susceptibility factor for atherosclerotic disease in the LDR setting.

## Supporting Information

S1 File Supporting information database file (2 excel sheets titled “TC and BCC study” and “Atherosclerosis study”). (XLSX)

S1 Table P-values obtained for the adjustment variables in the hereditary models analyzed in the thyroid and basal cell carcinoma study. (DOCX)

S2 Table P-values obtained for the adjustment variables in the hereditary models analyzed in the atherosclerosis study (whole cohort). (DOCX)

S3 Table P-values obtained for the adjustment variables in the hereditary models analyzed in the atherosclerosis study (non-irradiated group). (DOCX)

S4 Table P-values obtained for the adjustment variables in the hereditary models analyzed in the atherosclerosis study (irradiated group). (DOCX)
