# Supplementary material for: IL6-174 G>C Polymorphism (rs1800795) Association with Late Effects of Low Dose Radiation Exposure in the Portuguese Tinea Capitis Cohort
Source: PLoS One. 2016 Sep 23;11(9):e0163474. doi: 10.1371/journal.pone.0163474 (PMC5035001; doi:10.1371/journal.pone.0163474)
Supplement: S1 Table — (DOCX) [file pone.0163474.s002.docx]

S1 Table – P-values obtained for the adjustment variables in the hereditary models analyzed in the thyroid and basal cell carcinoma study.

| **Variable** | **Thyroid carcinoma** | | | **Basal cell carcinoma** | | |
| --- | --- | --- | --- | --- | --- | --- |
|  | Genotypic | Dominant | Recessive | Genotypic | Dominant | Recessive |
| **Gender** | 0.028 | 0.028 | 0.028 | 0.110 | 0.108 | 0.108 |
| **Age** | 0.374 | 0.371 | 0.373 | 0.003 | 0.003 | 0.003 |
