# Supplementary material for: IL6-174 G>C Polymorphism (rs1800795) Association with Late Effects of Low Dose Radiation Exposure in the Portuguese Tinea Capitis Cohort
Source: PLoS One. 2016 Sep 23;11(9):e0163474. doi: 10.1371/journal.pone.0163474 (PMC5035001; doi:10.1371/journal.pone.0163474)
Supplement: S2 Table — (DOCX) [file pone.0163474.s003.docx]

S2 Table – P-values obtained for the adjustment variables in the hereditary models analyzed in the atherosclerosis study (whole cohort).

| **Variable** | **Plaque presence** | | | **IMT** | | | **Stenosis** | | |
| --- | --- | --- | --- | --- | --- | --- | --- | --- | --- |
|  | Genotypic | Dominant | Recessive | Genotypic | Dominant | Recessive | Genotypic | Dominant | Recessive |
| **Gender** | 0.054 | 0.059 | 0.052 | 0.001 | 0.001 | 0.001 | 0.406 | 0.406 | 0.390 |
| **Age** | 0.004 | 0.006 | 0.004 | 0.040 | 0.059 | 0.043 | 0.016 | 0.016 | 0.014 |
| **Hypertension** | 0.002 | 0.001 | 0.002 | 0.152 | 0.122 | 0.136 | 0.007 | 0.007 | 0.008 |
| **Diabetes** | 0.005 | 0.007 | 0.006 | 0.371 | 0.370 | 0.339 | 0.140 | 0.139 | 0.164 |
| **Smoking habits** | 0.010 | 0.010 | 0.009 | 0.017 | 0.015 | 0.018 | 0.217 | 0.217 | 0.220 |
