# Supplementary material for: IL6-174 G>C Polymorphism (rs1800795) Association with Late Effects of Low Dose Radiation Exposure in the Portuguese Tinea Capitis Cohort
Source: PLoS One. 2016 Sep 23;11(9):e0163474. doi: 10.1371/journal.pone.0163474 (PMC5035001; doi:10.1371/journal.pone.0163474)
Supplement: S3 Table — (DOCX) [file pone.0163474.s004.docx]

S3 Table – P-values obtained for the adjustment variables in the hereditary models analyzed in the atherosclerosis study (non-irradiated group).

| **Variable** | **Plaque presence** | | | **IMT** | | | **Stenosis** | | |
| --- | --- | --- | --- | --- | --- | --- | --- | --- | --- |
|  | Genotypic | Dominant | Recessive | Genotypic | Dominant | Recessive | Genotypic | Dominant | Recessive |
| **Gender** | 0.836 | 0.832 | 0.820 | 0.585 | 0.558 | 0.705 | 0.080 | 0.080 | 0.080 |
| **Age** | 0.087 | 0.094 | 0.088 | 0.278 | 0.442 | 0.285 | 0.098 | 0.097 | 0.097 |
| **Hypertension** | 0.005 | 0.005 | 0.004 | 0.990 | 0.994 | 0.827 | 0.034 | 0.034 | 0.034 |
| **Diabetes** | 0.045 | 0.045 | 0.045 | 0.064 | 0.046 | 0.035 | 0.575 | 0.574 | 0.577 |
| **Smoking habits** | 0.004 | 0.004 | 0.004 | 0.098 | 0.110 | 0.059 | 0.044 | 0.044 | 0.043 |
