# Supplementary material for: IL6-174 G>C Polymorphism (rs1800795) Association with Late Effects of Low Dose Radiation Exposure in the Portuguese Tinea Capitis Cohort
Source: PLoS One. 2016 Sep 23;11(9):e0163474. doi: 10.1371/journal.pone.0163474 (PMC5035001; doi:10.1371/journal.pone.0163474)
Supplement: S4 Table — (DOCX) [file pone.0163474.s005.docx]

S4 Table – P-values obtained for the adjustment variables in the hereditary models analyzed in the atherosclerosis study (irradiated group).

| **Variable** | **Plaque presence** | | | **IMT** | | | **Stenosis** | | |
| --- | --- | --- | --- | --- | --- | --- | --- | --- | --- |
|  | Genotypic | Dominant | Recessive | Genotypic | Dominant | Recessive | Genotypic | Dominant | Recessive |
| **Gender** | 0.011 | 0.013 | 0.011 | <0.001 | <0.001 | <0.001 | 0.022 | 0.022 | 0.020 |
| **Age** | 0.018 | 0.021 | 0.016 | 0.052 | 0.055 | 0.047 | 0.037 | 0.036 | 0.026 |
| **Hypertension** | 0.065 | 0.043 | 0.070 | 0.091 | 0.075 | 0.097 | 0.058 | 0.058 | 0.068 |
| **Diabetes** | 0.040 | 0.053 | 0.041 | 0.697 | 0.680 | 0.675 | 0.200 | 0.198 | 0.232 |
| **Smoking habits** | 0.381 | 0.374 | 0.333 | 0.043 | 0.134 | 0.131 | 0.985 | 0.981 | 0.922 |
